# 2,4,5-Tri-4-pyridyl-1*H*-imidazole monohydrate

**DOI:** 10.1107/S1600536809032267

**Published:** 2009-09-05

**Authors:** Qiang Huang

**Affiliations:** a College of Mechanical and Materials Engineering, Jiujiang University, 332005 Jiujiang, JiangXi, People’s Republic of China

## Abstract

The title compound, C_18_H_13_N_5_·H_2_O, was synthesized by the condensation of pyridine-4-carbaldehyde and ammonium acetate, forming a multipyridyl ligand. In the crystal, mol­ecules are linked into chains by O—H⋯N hydrogen bonds. The chains are linked by weak C—H⋯N inter­actions, generating a layer structure.

## Related literature

2,4,5-Tri-4-pyrid­yl-imidazole is used in the construction of metal-organic coordination polymers, see: Liang *et al.* (2009 ▶).
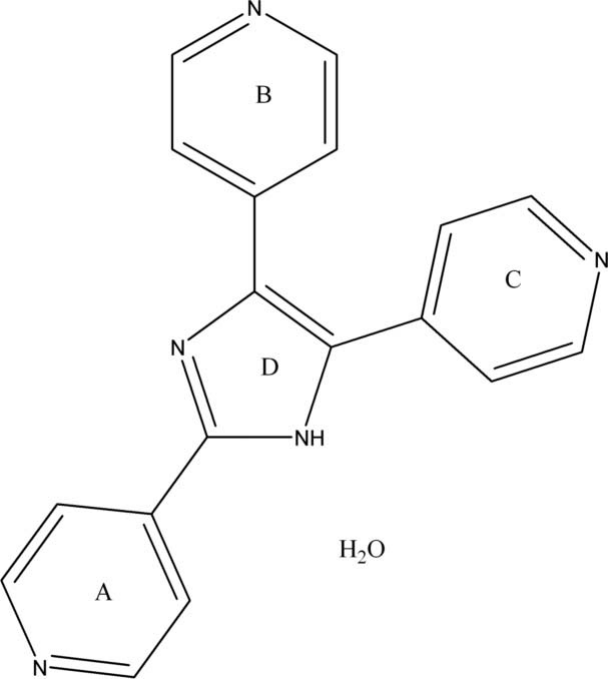


## Experimental

### 

#### Crystal data

C_18_H_13_N_5_·H_2_O
*M* *_r_* = 317.35
Triclinic, 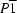

*a* = 8.910 (2) Å
*b* = 9.401 (2) Å
*c* = 10.638 (2) Å
α = 72.027 (4)°
β = 70.624 (4)°
γ = 77.716 (4)°
*V* = 793.4 (3) Å^3^
*Z* = 2
Mo *K*α radiation
μ = 0.09 mm^−1^
*T* = 293 K
0.30 × 0.26 × 0.22 mm

#### Data collection

Bruker APEXII area-detector diffractometer
Absorption correction: multi-scan (*SADABS*; Bruker, 2004 ▶) *T*_min_ = 0.974, *T*_max_ = 0.981
4313 measured reflections
3067 independent reflections
1720 reflections with *I* > 2σ(*I*)
*R*_int_ = 0.025

#### Refinement

*R*[*F* ^2^ > 2σ(*F* ^2^)] = 0.045
*wR*(*F* ^2^) = 0.059
*S* = 1.02
3067 reflections
217 parameters
3 restraints
H-atom parameters constrained
Δρ_max_ = 0.15 e Å^−3^
Δρ_min_ = −0.15 e Å^−3^

### 

Data collection: *APEX2* (Bruker, 2004 ▶); cell refinement: *SAINT* (Bruker, 2004 ▶); data reduction: *SAINT*; program(s) used to solve structure: *SHELXS97* (Sheldrick, 2008 ▶); program(s) used to refine structure: *SHELXL97* (Sheldrick, 2008 ▶); molecular graphics: *SHELXL97*; software used to prepare material for publication: *SHELXL97* and *publCIF* (Westrip, 2009 ▶).

## Supplementary Material

Crystal structure: contains datablocks I, global. DOI: 10.1107/S1600536809032267/hg2553sup1.cif

Structure factors: contains datablocks I. DOI: 10.1107/S1600536809032267/hg2553Isup2.hkl

Additional supplementary materials:  crystallographic information; 3D view; checkCIF report

## Figures and Tables

**Table 1:** Selected torsion angles (°)

C4—C3—C6—N2	−12.2 (4)
C14—C7—C8—C9	1.8 (5)
N1—C8—C9—C13	−88.5 (3)
N2—C7—C14—C18	−7.7 (3)

**Table 2:** Hydrogen-bond geometry (Å, °)

*D*—H⋯*A*	*D*—H	H⋯*A*	*D*⋯*A*	*D*—H⋯*A*
N1—H1*C*⋯O1	0.94	1.82	2.756 (2)	173
C10—H10⋯N2^i^	0.93	2.59	3.467 (3)	158
O1—H1*B*⋯N4^ii^	0.91	1.96	2.869 (2)	174
O1—H1*A*⋯N5^iii^	0.87	1.94	2.808 (2)	174

Symmetry codes: (i) 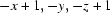; (ii) 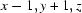; (iii) 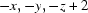.

**Fig. 1.**
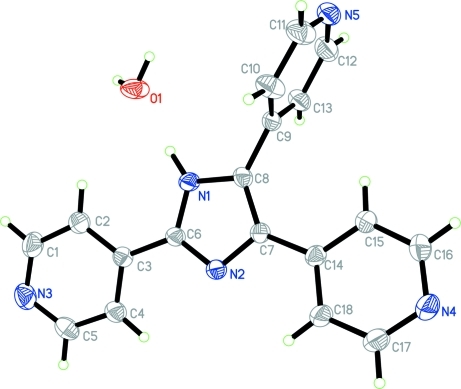
The molecular structure of (I), showing 30% probability displacement ellipsoids.

**Fig. 2.**
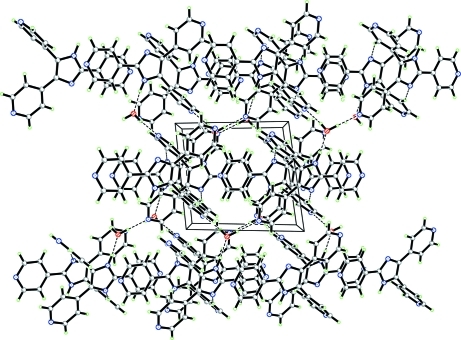
The packing diagram of (I), viewed along the c axis; hydrogen bonds are shown as dashed lines.

## supplementary crystallographic information

### Comment

2,4,5-tri(4-pyridyl)imidazole is a multipyridyl compound, which is useful to construct new metal-organic coordination polymers (Liang *et al.*, 2009). In this paper, we report the synthesis and X-ray crystal structure analysis of the title compound, (I,) 2,4,5-tri(4-pyridyl)imidazole with one co-crystallized water molecule.

In 2,4,5-tri(4-pyridyl)imidazole three pyridyl groups are directly connected with the imidazole ring. The dihedral angles between the mean planes of pyridyl ring A and imidazole ring D is 11.6 (4)°, that of pyridyl ring B and imidazole ring D is 8.4 (3)°, and that of pyridyl ring C and imidazole ring D is 84.1 (3)°, suggesting that the plane of ring A and B are co-planar with ring D, but that ring C and ring D are almost vertical.

In the crystal lattice the molecules are linked by O—H···N hydrogen bonds, and by weak C—H···N interactions to generate a three-dimensional layer structure (Fig 2).

### Experimental

A mixture of 2 g (0.018 mol) of 4-pyridinecarbaldehyde and 8 g (0.1 mol) of ammonium acetate was heated to 393 K with stirring 3 h. The reaction mixture was cooled, the precipitate was filtered off, washed with water, 5% solution of NaOH, and recrystallized from ethanol. Single crystals of 2,4,5-tri(4-pyridyl)imidazole suitable for X-ray analysis were obtained by slow evaporation at room temperature of a methanol solution. ^1^H NMR (500 MHz, DMSO-d_6_) 8.70(t, 4H), 8.54 (s, 2H), 8.02 (s, 2H), 7.53 (s, 4H) MS: found [*M*_+_] = 299.1, cal [*M*_+_] = 299.3.

### Refinement

The H atoms of the pyridyl rings were constrainted as idealized aromatic CH groups. The H atoms of water, H1A and H1B, were located in a difference Fourier map and the O1—H1A and O1—H1B were restrained to 0.85Å, the H1A—H1Bwas restrained to 1.35Å. The proton on the imidazole N atom, H1C,was also located in a difference Fourier map and N1—H1C was restrained to 0.94Å. The U_iso_(H) was equal to 1.2 times that of the parent atoms for all H atoms.

### Crystal data

**Table:**

C_18_H_13_N_5_·H_2_O	*Z* = 2
*M**_r_* = 317.35	*F*(000) = 332
Triclinic, *P*1	*D*_x_ = 1.328 Mg m^−^^3^
Hall symbol: -P 1	Mo *K*α radiation, λ = 0.71073 Å
*a* = 8.910 (2) Å	Cell parameters from 4826 reflections
*b* = 9.401 (2) Å	θ = 0.9–28.3°
*c* = 10.638 (2) Å	µ = 0.09 mm^−^^1^
α = 72.027 (4)°	*T* = 293 K
β = 70.624 (4)°	Block, yellow
γ = 77.716 (4)°	0.30 × 0.26 × 0.22 mm
*V* = 793.4 (3) Å^3^	

### Data collection

**Table:**

Bruker APEXII area-detector diffractometer	3067 independent reflections
Radiation source: fine-focus sealed tube	1720 reflections with *I* > 2σ(*I*)
graphite	*R*_int_ = 0.025
φ and ω scans	θ_max_ = 26.1°, θ_min_ = 2.3°
Absorption correction: multi-scan (*SADABS*; Bruker, 2004)	*h* = −11→10
*T*_min_ = 0.974, *T*_max_ = 0.981	*k* = −11→8
4313 measured reflections	*l* = −13→12

### Refinement

**Table:**

Refinement on *F*^2^	Primary atom site location: structure-invariant direct methods
Least-squares matrix: full	Secondary atom site location: difference Fourier map
*R*[*F*^2^ > 2σ(*F*^2^)] = 0.045	Hydrogen site location: inferred from neighbouring sites
*wR*(*F*^2^) = 0.059	H-atom parameters constrained
*S* = 1.02	*w* = 1/[σ^2^(*F*_o_^2^) + (0.0004*P*)^2^] where *P* = (*F*_o_^2^ + 2*F*_c_^2^)/3
3067 reflections	(Δ/σ)_max_ < 0.001
217 parameters	Δρ_max_ = 0.15 e Å^−^^3^
3 restraints	Δρ_min_ = −0.15 e Å^−^^3^

### Special details

**Table:**

Geometry. All e.s.d.'s (except the e.s.d. in the dihedral angle between two l.s. planes) are estimated using the full covariance matrix. The cell e.s.d.'s are taken into account individually in the estimation of e.s.d.'s in distances, angles and torsion angles; correlations between e.s.d.'s in cell parameters are only used when they are defined by crystal symmetry. An approximate (isotropic) treatment of cell e.s.d.'s is used for estimating e.s.d.'s involving l.s. planes.
Refinement. Refinement of *F*^2^ against ALL reflections. The weighted *R*-factor *wR* and goodness of fit *S* are based on *F*^2^, conventional *R*-factors *R* are based on *F*, with *F* set to zero for negative *F*^2^. The threshold expression of *F*^2^ > σ(*F*^2^) is used only for calculating *R*-factors(gt) *etc*. and is not relevant to the choice of reflections for refinement. *R*-factors based on *F*^2^ are statistically about twice as large as those based on *F*, and *R*- factors based on ALL data will be even larger.

### Fractional atomic coordinates and isotropic or equivalent isotropic displacement parameters (Å^2^)

**Table:**

	*x*	*y*	*z*	*U*_iso_*/*U*_eq_	
C1	0.3675 (3)	0.6040 (2)	0.2766 (3)	0.0664 (8)	
H1	0.2702	0.6621	0.2711	0.080*	
C2	0.3638 (3)	0.4768 (2)	0.3848 (2)	0.0539 (7)	
H2	0.2669	0.4515	0.4492	0.065*	
C3	0.5060 (3)	0.3871 (2)	0.3965 (2)	0.0403 (6)	
C4	0.6444 (3)	0.4344 (2)	0.2983 (2)	0.0505 (7)	
H4	0.7436	0.3798	0.3023	0.061*	
C5	0.6343 (3)	0.5632 (3)	0.1943 (2)	0.0616 (8)	
H5	0.7295	0.5920	0.1291	0.074*	
C6	0.5121 (2)	0.2487 (2)	0.5058 (2)	0.0391 (6)	
C7	0.5929 (2)	0.0342 (2)	0.6266 (2)	0.0394 (6)	
C8	0.4352 (2)	0.0717 (2)	0.6957 (2)	0.0409 (6)	
C9	0.3254 (2)	−0.0054 (2)	0.8263 (2)	0.0411 (6)	
C10	0.2358 (3)	−0.1086 (2)	0.8270 (3)	0.0599 (8)	
H10	0.2408	−0.1288	0.7453	0.072*	
C11	0.1383 (3)	−0.1815 (3)	0.9518 (3)	0.0648 (8)	
H11	0.0798	−0.2522	0.9513	0.078*	
C12	0.2082 (3)	−0.0560 (3)	1.0683 (3)	0.0626 (8)	
H12	0.1995	−0.0363	1.1511	0.075*	
C13	0.3095 (2)	0.0224 (2)	0.9487 (2)	0.0530 (7)	
H13	0.3659	0.0932	0.9519	0.064*	
C14	0.7046 (2)	−0.1000 (2)	0.6596 (2)	0.0408 (6)	
C15	0.6737 (2)	−0.2114 (2)	0.7826 (2)	0.0518 (7)	
H15	0.5777	−0.2024	0.8512	0.062*	
C16	0.7863 (3)	−0.3359 (2)	0.8028 (3)	0.0609 (8)	
H16	0.7620	−0.4090	0.8862	0.073*	
C17	0.9555 (3)	−0.2501 (3)	0.5936 (3)	0.0675 (9)	
H17	1.0532	−0.2614	0.5274	0.081*	
C18	0.8497 (2)	−0.1228 (2)	0.5640 (2)	0.0558 (8)	
H18	0.8765	−0.0523	0.4792	0.067*	
N1	0.38651 (19)	0.20619 (17)	0.61671 (17)	0.0441 (5)	
H1C	0.2849	0.2595	0.6454	0.053*	
N2	0.63900 (18)	0.14695 (18)	0.50748 (17)	0.0417 (5)	
N3	0.4987 (3)	0.6500 (2)	0.1798 (2)	0.0669 (7)	
N4	0.9263 (2)	−0.3581 (2)	0.7116 (2)	0.0642 (7)	
N5	0.1230 (2)	−0.1573 (2)	1.0716 (2)	0.0578 (6)	
O1	0.07758 (16)	0.34217 (15)	0.70240 (16)	0.0723 (6)	
H1A	0.0112	0.2912	0.7740	0.087*	
H1B	0.0296	0.4357	0.7119	0.087*	

### Atomic displacement parameters (Å^2^)

**Table:**

	*U*^11^	*U*^22^	*U*^33^	*U*^12^	*U*^13^	*U*^23^
C1	0.0606 (18)	0.0525 (17)	0.075 (2)	−0.0030 (13)	−0.0288 (16)	0.0057 (15)
C2	0.0493 (16)	0.0470 (16)	0.0557 (18)	−0.0069 (12)	−0.0138 (13)	−0.0002 (13)
C3	0.0441 (14)	0.0339 (13)	0.0413 (15)	−0.0052 (11)	−0.0110 (12)	−0.0087 (11)
C4	0.0506 (15)	0.0445 (15)	0.0472 (16)	−0.0069 (12)	−0.0090 (13)	−0.0039 (13)
C5	0.0673 (19)	0.0564 (17)	0.0499 (18)	−0.0210 (14)	−0.0076 (15)	0.0009 (14)
C6	0.0371 (14)	0.0352 (13)	0.0391 (15)	−0.0044 (11)	−0.0068 (12)	−0.0059 (11)
C7	0.0389 (14)	0.0316 (13)	0.0431 (15)	−0.0020 (10)	−0.0094 (11)	−0.0072 (12)
C8	0.0427 (14)	0.0306 (13)	0.0403 (15)	−0.0023 (11)	−0.0074 (12)	−0.0029 (11)
C9	0.0379 (14)	0.0310 (14)	0.0423 (16)	0.0039 (10)	−0.0062 (12)	−0.0035 (12)
C10	0.0671 (18)	0.0481 (16)	0.0545 (18)	−0.0157 (13)	0.0014 (14)	−0.0138 (14)
C11	0.0677 (19)	0.0475 (17)	0.069 (2)	−0.0186 (13)	−0.0037 (17)	−0.0105 (16)
C12	0.0691 (19)	0.0614 (18)	0.0475 (18)	−0.0080 (14)	−0.0121 (15)	−0.0054 (15)
C13	0.0559 (17)	0.0472 (16)	0.0466 (17)	−0.0126 (12)	−0.0087 (13)	−0.0017 (13)
C14	0.0371 (13)	0.0352 (13)	0.0478 (16)	−0.0029 (10)	−0.0111 (12)	−0.0097 (12)
C15	0.0468 (15)	0.0437 (15)	0.0510 (17)	0.0046 (12)	−0.0081 (13)	−0.0060 (13)
C16	0.0644 (18)	0.0460 (16)	0.0592 (19)	0.0018 (14)	−0.0175 (15)	−0.0014 (13)
C17	0.0456 (16)	0.0552 (18)	0.077 (2)	0.0033 (13)	−0.0018 (15)	−0.0065 (16)
C18	0.0424 (14)	0.0429 (15)	0.0631 (18)	0.0009 (12)	−0.0061 (13)	−0.0009 (13)
N1	0.0363 (11)	0.0334 (11)	0.0475 (13)	0.0033 (8)	−0.0024 (10)	−0.0050 (10)
N2	0.0382 (11)	0.0356 (11)	0.0430 (12)	−0.0028 (9)	−0.0068 (9)	−0.0048 (9)
N3	0.0751 (16)	0.0556 (14)	0.0610 (16)	−0.0149 (13)	−0.0254 (13)	0.0083 (12)
N4	0.0509 (14)	0.0481 (13)	0.0766 (17)	0.0070 (10)	−0.0139 (12)	−0.0061 (12)
N5	0.0524 (14)	0.0451 (14)	0.0571 (16)	−0.0041 (10)	−0.0046 (12)	−0.0001 (12)
O1	0.0484 (10)	0.0450 (10)	0.0835 (13)	0.0044 (7)	0.0126 (9)	−0.0020 (9)

### Geometric parameters (Å, °)

**Table:**

C1—N3	1.325 (2)	C10—H10	0.9300
C1—C2	1.377 (3)	C11—N5	1.321 (3)
C1—H1	0.9300	C11—H11	0.9300
C2—C3	1.385 (3)	C12—N5	1.324 (3)
C2—H2	0.9300	C12—C13	1.385 (3)
C3—C4	1.379 (2)	C12—H12	0.9300
C3—C6	1.458 (3)	C13—H13	0.9300
C4—C5	1.374 (3)	C14—C18	1.377 (2)
C4—H4	0.9300	C14—C15	1.386 (3)
C5—N3	1.333 (3)	C15—C16	1.382 (3)
C5—H5	0.9300	C15—H15	0.9300
C6—N2	1.318 (2)	C16—N4	1.324 (2)
C6—N1	1.352 (2)	C16—H16	0.9300
C7—C8	1.379 (2)	C17—N4	1.335 (3)
C7—N2	1.381 (2)	C17—C18	1.380 (3)
C7—C14	1.460 (3)	C17—H17	0.9300
C8—N1	1.359 (2)	C18—H18	0.9300
C8—C9	1.480 (3)	N1—H1C	0.9393
C9—C13	1.362 (3)	O1—H1A	0.8697
C9—C10	1.378 (3)	O1—H1B	0.9124
C10—C11	1.383 (3)		
			
N3—C1—C2	125.1 (2)	N5—C11—H11	117.9
N3—C1—H1	117.4	C10—C11—H11	117.9
C2—C1—H1	117.4	N5—C12—C13	123.8 (3)
C1—C2—C3	119.2 (2)	N5—C12—H12	118.1
C1—C2—H2	120.4	C13—C12—H12	118.1
C3—C2—H2	120.4	C9—C13—C12	119.2 (2)
C4—C3—C2	116.7 (2)	C9—C13—H13	120.4
C4—C3—C6	120.74 (19)	C12—C13—H13	120.4
C2—C3—C6	122.61 (19)	C18—C14—C15	116.15 (19)
C5—C4—C3	119.3 (2)	C18—C14—C7	119.69 (19)
C5—C4—H4	120.3	C15—C14—C7	124.15 (19)
C3—C4—H4	120.3	C16—C15—C14	119.7 (2)
N3—C5—C4	125.0 (2)	C16—C15—H15	120.1
N3—C5—H5	117.5	C14—C15—H15	120.1
C4—C5—H5	117.5	N4—C16—C15	124.4 (2)
N2—C6—N1	111.24 (18)	N4—C16—H16	117.8
N2—C6—C3	124.49 (18)	C15—C16—H16	117.8
N1—C6—C3	124.24 (18)	N4—C17—C18	124.0 (2)
C8—C7—N2	109.24 (17)	N4—C17—H17	118.0
C8—C7—C14	130.29 (19)	C18—C17—H17	118.0
N2—C7—C14	120.44 (17)	C14—C18—C17	120.1 (2)
N1—C8—C7	105.72 (17)	C14—C18—H18	119.9
N1—C8—C9	121.56 (17)	C17—C18—H18	119.9
C7—C8—C9	132.71 (19)	C6—N1—C8	108.05 (16)
C13—C9—C10	118.0 (2)	C6—N1—H1C	129.5
C13—C9—C8	121.6 (2)	C8—N1—H1C	122.1
C10—C9—C8	120.4 (2)	C6—N2—C7	105.72 (16)
C9—C10—C11	118.5 (2)	C1—N3—C5	114.6 (2)
C9—C10—H10	120.7	C16—N4—C17	115.58 (19)
C11—C10—H10	120.7	C11—N5—C12	116.2 (2)
N5—C11—C10	124.3 (3)	H1A—O1—H1B	96.8
			
N3—C1—C2—C3	−0.1 (4)	C8—C7—C14—C18	170.3 (3)
C1—C2—C3—C4	1.3 (4)	N2—C7—C14—C18	−7.7 (3)
C1—C2—C3—C6	−178.4 (2)	C8—C7—C14—C15	−8.4 (4)
C2—C3—C4—C5	−1.5 (3)	N2—C7—C14—C15	173.6 (2)
C6—C3—C4—C5	178.2 (2)	C18—C14—C15—C16	0.0 (4)
C3—C4—C5—N3	0.5 (4)	C7—C14—C15—C16	178.7 (2)
C4—C3—C6—N2	−12.2 (4)	C14—C15—C16—N4	0.3 (4)
C2—C3—C6—N2	167.5 (2)	C15—C14—C18—C17	−0.6 (4)
C4—C3—C6—N1	169.8 (2)	C7—C14—C18—C17	−179.4 (2)
C2—C3—C6—N1	−10.4 (4)	N4—C17—C18—C14	0.9 (4)
N2—C7—C8—N1	0.8 (3)	N2—C6—N1—C8	1.3 (3)
C14—C7—C8—N1	−177.4 (2)	C3—C6—N1—C8	179.5 (2)
N2—C7—C8—C9	180.0 (2)	C7—C8—N1—C6	−1.3 (3)
C14—C7—C8—C9	1.8 (5)	C9—C8—N1—C6	179.5 (2)
N1—C8—C9—C13	−88.5 (3)	N1—C6—N2—C7	−0.8 (3)
C7—C8—C9—C13	92.5 (3)	C3—C6—N2—C7	−179.0 (2)
N1—C8—C9—C10	91.7 (3)	C8—C7—N2—C6	0.0 (3)
C7—C8—C9—C10	−87.4 (4)	C14—C7—N2—C6	178.4 (2)
C13—C9—C10—C11	−1.8 (3)	C2—C1—N3—C5	−0.9 (4)
C8—C9—C10—C11	178.05 (19)	C4—C5—N3—C1	0.7 (4)
C9—C10—C11—N5	1.1 (4)	C15—C16—N4—C17	0.0 (4)
C10—C9—C13—C12	1.6 (3)	C18—C17—N4—C16	−0.6 (4)
C8—C9—C13—C12	−178.25 (19)	C10—C11—N5—C12	0.0 (4)
N5—C12—C13—C9	−0.6 (4)	C13—C12—N5—C11	−0.2 (4)

### Hydrogen-bond geometry (Å, °)

**Table:**

*D*—H···*A*	*D*—H	H···*A*	*D*···*A*	*D*—H···*A*
N1—H1C···O1	0.94	1.82	2.756 (2)	173
C10—H10···N2^i^	0.93	2.59	3.467 (3)	158
O1—H1B···N4^ii^	0.91	1.96	2.869 (2)	174
O1—H1A···N5^iii^	0.87	1.94	2.808 (2)	174

Symmetry codes: (i) −*x*+1, −*y*, −*z*+1; (ii) *x*−1, *y*+1, *z*; (iii) −*x*, −*y*, −*z*+2.
